# Information needs assessment of medical equipment offices based on Critical Success Factors (CSF) and Business System Planning (BSP) methods

**Published:** 2015

**Authors:** F Khorrami, M Ahmadi, A Alizadeh, N Roozbeh, S Mohseni

**Affiliations:** * Department of Health Information Technology, Hormozgan University of Medical Sciences, Bandar Abbas, Iran; ** Department of Health Information Management, Tehran University of Medical Sciences, Tehran, Iran; *** Department of Public Health, Faculty of Health, Hormozgan University of Medical Sciences, Bandar Abbas, Iran; **** Reproductive Health, Shahid Beheshti University of Medical Science, Tehran, Iran

**Keywords:** needs assessment, Management Information Systems, medical equipment and supplies, medical, equipment office

## Abstract

**Introduction:** Given the ever-increasing importance and value of information, providing the management with a reliable information system, which can facilitate decision-making regarding planning, organization and control, is vitally important. This study aimed to analyze and evaluate the information needs of medical equipment offices.

**Methods:** This descriptive applied cross-sectional study was carried out in 2010. The population of the study included the managers of statistic and medical records at the offices of vice-chancellor for treatment in 39 medical universities in Iran. Data were collected by using structured questioners. With regard to different kinds of designing information systems, sampling was done by two methods, BSP (based on processes of job description) and CSF method (based on critical success factors). The data were analyzed by SPSS-16.

**Results:** Our study showed that 41% of information needs were found to be critical success factors of managers of office. The first priority of managers was “the number of bed and bed occupancy in hospitals”. Of 29 identified information needs, 62% were initial information needs of managers (from the viewpoints of managers). Of all, 4% of the information needs were obtained through the form, 14% through both the form and database, 11% through the web site, and 71% had no sources (forms, databases, web site).

**Conclusion:** Since 71% of the information needs of medical equipment offices managers had no information sources, the development of information system in these offices seems to be necessary. Despite the important role of users in designing the information systems (identifying 62% of information needs), other scientific methods is also needed to be utilized in designing the information systems.

## Introduction

Medical devices developed for human application are used for diagnostic or treatment purposes. They may be either an instrument, an apparatus, or a material. Moreover, these devices can be used for daily patient care as well as for medical scientific purposes [**1**]. These medical devices must be kept in good condition to prevent from injuries occurring both in patients as well as in users. Moreover, to face the tough competition environment and complex health care system, the hospital should take the appropriate cost controls in response to that situation. The clinical engineering department (CED) in the hospital is responsible for the patient and clinical staff safety in using medical devices. Besides, the cost control in related operational activities of medical devices (such as purchase, contract, repair, and maintenance) is another important job for this department [**2**-**5**].

In Iran, CED is known as the medical equipments in the organizational structure of the Ministry of Health Department treatment and hospitals.

Medical equipment office (MEO) is one of the most important offices among hygiene ministry level and the country’s medical sciences universities. The main responsibility of this office in the hygiene ministry level is to organize the medical equipment condition in the country and the supervising on production process, importing, distributing, supplying, using, maintaining, exporting, quality, and quantity improving the country’s internal medical equipment producing and supervising the importing of medical equipment quality. Finding needs of medical equipment in the country, getting an ID for the medical equipment firms, coding and dividing this equipment, controlling this equipment in the market, evaluating complains and referring them to the judiciary office are important tasks of this center [**6**].

Nowadays, in developing countries, half of the medical equipment are hardly in a proper situation for using and because of the medical equipment without an appropriate management (improper infrastructure, without necessary foundation for purchasing and providing, training and maintenance and operation from imported technology) wasting national hygiene source are still being continued [**7**].

On the other hand, with concern to the presence limits in human and financial sources especially in the governmental part, protecting from the national investment and with providing an appropriate sufficiency vital role of medical equipment, are the other responsibilities of the ministry and other centers [**5**,**8**,**9**].

According to the estimation done in Iran, for launching new hospitals, one third of the construction costs and equipping hospital are allocated to the medical equipment purchasing and on the other hand, for medical equipment repair and maintenance; 10-20% of the purchasing price should be estimated in the budget in advance [**10**]. In other words, if the hospital’s chief believes in the main plan of medical equipment management and use, there would be economy in the hospital’s costs, in a way that only by doing one of the maintenance management details meaning preventing maintenance there can be a reduction in repair cost from 45 to 50% [**7**,**10**,**11**]. So, the medical equipment case in all governmental parts, cooperative, private in management and economical view are of important concern and not paying attention to those would make managers face serious challenges [**10**].

According to the mentioned problems and this view that “each information always and in each place should be accessible for managers” would force information managers use scientific methods for providing information needs of this office [**12**], since the gathered information could instantly help the medical equipment managers in the decision-making. In order to identify the information needs, sample of information systems should be followed, that besides basic conditions and projects operating standards, the user’s urgent and primary needs should be concerned [**13**]. In this research with an emphasis and contingency view, the samples were done with the help of two methods, BSP (Business System Planning) and CSF (Critical Success Factors) to information needs analyzing the medical equipment office.

**BSP method:** This methodology in the 70s was invented to solve problems and disorders relevant to the information systems in the IBM Corporation and were gradually improved. Until present, it has become a successful method for different projects execution in this field (either in industry or in services) [**14**]. This constructive method mentioned in the information systems scheduling discussion, for assisting the organization in information systems planning, because the user’s information needing security was utilized from success factors of this methodology, concern and emphasis on occupation processes, data management being one of the most important of the organization sources, planning from top to bottom and the system’s bottom to top [**15**].

**CSF method:** The idea of identifying critical success factors as a basis for determining the information needs of managers was proposed by Daniel [**11**], but popularized by Rockart [**19**]. The idea is very simple: in any organization, certain factors will be critical to the success of that organization, in the sense that, if objectives associated with the factors are not achieved, the organization will fail - perhaps catastrophically. The CSFs approach was applied in case studies carried out in the UK universities [**16**].

In this research, researchers tried to evaluate and analyze hygiene information needs, and medical equipment office Iran University of Medical Sciences, so, hopefully, the results of this research could be utilized in designing and managing the information choice in this office.

**Working method**

This research is of functional and descriptive type. Study societies in this research were department managers of medical equipment of 39 of the medical universities of the country, and were done in 2010. The questionnaires were given to the whole research society persons, by sending an administrable e-envelope or e-mail and the answers were taken from 14 universities after 3 times following and during 8 months

According to the variety of methods in information systems design, this research was sampled by, BSP method based on processes and responsibility explanations and CFS method based on vital factors of successful managers. The reason for choosing 2 methods at the same time was that despite the other researcher’s hint, since the differences in organizations information needs and this point that organizations are in different steps in information systems life cycle, it cannot be said that there is a fixed methodology for all the information systems design of organization management. Having said that it has been proposed that the information systems design should be done according to the contingency view and with one or more of the methods.

This research was done in 4 stages. At first, it is evaluated by the open questionnaire, the need of remedy hygiene information from the approach of occupied managers in the medical equipment section of remedy assistance on medical sciences university of the country. With the use of this questionnaire, the manager’s information was gathered in two sections (demographic information) and variety of information needs.

In the second stage, it was evaluated by internal and external sources, infrastructure, responsibility explanations, and processes relevant to the section and information defined for managers for dealing with the responsibility. Then, in the third stage, with the use of the results from the previous stages, a questionnaire was defined including all the managers information needs of medical equipment office of remedy assistance. This questionnaire was divided into 4 categories of general information, specific information, human and resource information and references to laws, regulations and strategic documents for defining priority, being given to the managers’ hand and experts with experience in the medical equipment office of medical sciences universities of the country, so as to divide certified information needs according to Likert rule (from very high 5 to poor 1). The prioritization of information needs in this stage was specified by the average equilibrium. In cases that average would be equal, the first one forth and in case of the first one forth they were equal, the quantity of frequency with high and very high priority, in priority of needs would be picked as a proper one. The proper choice for information need was the first one forth. Based on that, if the first one forth was 3 or more than that, (in other words more than 25% of the managers, the priority of needs had been picked less than average). The relevant information need was accounted as a proper level and otherwise as an improper one. In the fourth stage, after prioritizing and defining the information needs, source of getting these needs in the existence system, was defined and the best source for getting information needs was proposed. In order to analyze data, SPSS software V.16 was utilized.

## Results

72% were study category managers in the range of 30 to 39 years old. 71% were men and 29% women. 28% were unit managers of medical engineering field, 36% electronics engineering, and 36% other fields and their average management experience was of 5 years. The following findings were based on 4 categorized groups of information needs and they are summarized in **Table 1** according to the research goals:

**Table 1:** Total number/ percent of information needs in groups and the source of preparation of information needs

The title of group	Total number of information needs	The most primitive group information needs	The certified information needs based on managers view (percent)	The source of preparation of information needs			
	Abundance (percent)			The Manual forms	The data bank	Web site	The lack of sources
General information group	7 (44%)	The available units and proficiencies in hospitals	86%	14%	57%	-	29%
Specific information group	16 (55%)	The list of benedictory machines and equipment by vindication	50%	-	-	7%	93%
Human resource information group	4 (12%)	The number of equipment experts man power employed in hospitals	75%	-	-	-	100%
References to laws, Regulations and Strategic documents information group	2 (7%)	None	50%	-	-	100%	-
Total/ The most primary group information needs among of groups	29 (100%)	The list of benedictory machines and equipment by vindication	62%	4%	14%	11%	71%

**General information group:** 86% of the needs of this group were included with primary information needs from managers’ approach. 43% of the needs of this group were included within the top ten priorities (CSF) of managers in MEO.

**Specific information group:** 50% of the needs of this group were included with primary information needs from the managers’ approach. 44% of the needs of this group were included within the top ten priorities of the office managers of medical equipment. Between this group’s specified information needs, “the done research designs of medical equipment field”, was the only information need below the proper range. “The appliances list and hospitals demand equipment region’s remedy hygiene center with persuasive demand” was the most prioritized in this group and in all the groups of the medical equipment office.

**Human resource information group:** 75% of the needs of this group were included with primary information needs from the manager’s approach. 25% of the needs of this group were included within the top ten priorities of the office managers of medical equipment.

**References to laws, Regulations, and Strategic documents information group:** one case of two in this group was included within the primary information needs from the manager’s approach. 

All in all, 62% of the group’s specified information needs included with primary information needs from the manager’s approach. 3% of the information needs were below the proper range. 4% of the information needs were accessible by manual forms, 14% by manual and electronic forms at the same time and 11% by an internet website and 71% was without any information sources such as form, internet website or database. 41% of these information needs of this unit included factors of managers with critical success **Table 2**.

**Table 2:** Critical success factors of managers in MEO

The number of primitive	Informational needs	Managers view (yes/ no)	The precedence indexes				precedence conclusion	
			Standard deviation	Average of privilege	Privileges total More toward high	Advantage total	number of primitive in group	number of primitive in group
1	The list of benedictory machines and equipment in hospital and curing-medicine centers by vindication	Yes	0.51	4.57	64	64	1	1
2	The regulation of medico equipment	Yes	0.65	4.5	60	63	1	2
2	Circulars and guidelines relating to the procurement of medical equipment	No	0.65	4.5	60	63	1	2
3	Medical Equipment ID along with beneficial machine age and seating side, device responsible and year of installation and so on	Yes	0.85	4.5	60	62	2	3
4	The manufacture data bank of active medico equipment in country	Yes	0.93	4.35	56	61	3	4
5	The number of medico equipment expert power employed in different hospital	Yes	0.97	4.21	51	59	2	5
6	reports of medical equipment purchasing expertise committee	Yes	0.8	4.21	50	59	4	6
7	The existence units in province hospitals	Yes	0.89	4.21	54	59	1	7
7	The existence expert in province hospitals	Yes	0.89	4.21	54	59	1	7
8	The committee proceedings of medico equipment	Yes	0.7	4.21	53	59	5	8
9	Guidance booklet of using devices user manual	Yes	0.95	4.14	50	58	6	9
10	The categorized information of hospitals and Para-clinic in order to adapt with solicitations	Yes	0.66	4.14	52	58	2	10

## Discussion

The goal of this study was to define and evaluate unit managers information needs of medical equipment of the medical sciences universities in the country. Our results demonstrated that 24% of the information needs are in general information group, 55% in specific information group, 14% in human resources group and 7% in References to laws, Regulations and Strategic documents information group; 71 percent of these information needs having been identified and having no information source, so that it showed the managers in decision time that they do not have access to 71 percent of information which they should have had. The first research goal was to determine the information needs from the manager’s approach, the results showing that 62% of the information needs were identified by the managers themselves in the first stage. The same researches about the design of information systems on user involvement and the creation of a data dictionary had a particularly strong emphasis on managers at different levels [**17**-**19**].

The second goal was to identify the information needs in MEO managers from other sources. In this stage of research, at the first structure, task works and processes in MEO were specified, because the responsibility explanation and structure and research goal were the most noticeable points in the BSP method. This way the analyzer could analyze the system details better and they could get enough information in the organization case and centers of information producing the next steps. 38% of the managers information needs were specified this way. In his research, Babaie stated that the reason for not taking the users’ needs seriously was that they tended to make the border for their needs and they only demanded for information that they were aware. That was why the library studies and the use of scientific methods for the identification of information needs was necessary, those being hidden in the managers approach [**20**].

The third research goal was to evaluate (prioritize) and define the information needs from the first and second stages. After prioritizing the information needs, 3% of the information needs turned out to be below the group from the prioritized level. Prioritizing is one of the uses of CSF method in this research. In this method, for each organization, something less than 10 critical or sensitive factors [**21**] that take into account the decision-making, were introduced. These factors depend on specific conditions of the organizations so they should be rectified during the experience period. These key factors needed to be consecutively considered by contractors. Critical success factors of MEO managers are shown in **Table 2**.

The most prioritized MEO information need was “The list of requested sets and equipments with justification”. According to the survey of the World Health Organization Global expenditure on medical devices increased from US$ 145 billion in 1998 to US$ 220 billion in 2006, representing an annual growth rate exceeding 10%. Surely, management of requests issue can help reducing costs and storage devices and also fixing the defective of centers which is covered by the storage program [**9**].

According to **Table 2**, the information need “medical equipment principles” and “directives” and instructions of providing and purchasing the medical equipments with little space, being the second priority of MEOs that this is covered by the website of the medical office of the Ministry of Health, that can be a sample for the other units so as to share information between colleagues and managers.

Information needs “medical equipment registration system with an appliance of useful life cycle and position, appliance responsible and the installation year” being the third priorities. In an article entitled “Effect of management system implementation and maintenance of medical equipment in VALIASR hospital costs in Arak 2006”, Jadidi underlined that after the evaluation of 691 medical equipment appliance, 57.73% of them were without technical certification and this factor was mentioned as a block in the optimal use of medical equipment [**10**].

Information needs “information database of current medical equipment companies in Iran” represents the fourth priority. In their article entitled “Materials Database Reorganization Plan, Medical Equipment Country” Khani and colleagues confirmed the importance of medical equipment certification and total information gathering relevant to the current companies in medical equipment and facilities including inside and outside, continuing that this information database creates high discipline in the medical equipment information structure. They also added that the most important benefit of having a certification of medical engineering companies is the awareness of the import range of each company, accessibility to medical equipment statistics efficiency condition especially in the economical equipment, but unfortunately, in offices, the current cycle relevant to the medical equipment, there is no organized system for CVs of companies, branches and importers. This issue caused that sometimes, based on the expert’s personal information, managers would have unexpected functions relevant to this issue [**22**].

The fifth priority relevant to the information need was “Specifications of manpower who is employed in the medical centers”. Based on the evaluation performed by Amerion, 44% of the existent medical equipment does not have even one technician or engineer [**23**]. Also in the same study, after the evaluation of 691 medical equipment appliances, Jadidi declared that 74% of the appliances do not have fixed and experienced human forces and this is mentioned as the optimal use block of the medical equipment [**10**]. 

The ninth information need was “guideline paper of appliance uses“. In their article entitled “The study of maintenance and care of the cost of medical equipment to hospitals Iranian health Medical Sciences University 2000”, Noori and colleagues showed that only 37.2% of the under study hospitals used guideline paper from the medical equipment and 15.6% of the under study equipment had service guideline paper. These papers showed service process and efficient detection tables of medical appliances and consecutive detecting date that should be followed by a caring unit and medical engineering or companies with after sale services and mentioned cases, so checking and detecting services of this appliance are done better [**6**].

Information needs “Sectors and professions in hospitals” and “hospital leveling information and Para clinics for adjusting with demands” are the seventh and ninth priorities in the critical success factors table, including prioritized general information of the medical equipment managers, that play an important role in the proper decision for the purchasing of the needed medical equipment for under cover centers [**24**]. 

The fourth goal of the research was the proposal for source or sources of prioritized information needs of remedy assistance medical equipment unit. 71% of the information needs was without specific sources such as manual form, website or software.

## Conclusion

Based on the results that were mentioned, it is recommended that: since the information need study does not have that much background in Iran and according to the done searching in this case, the product of these studies are limited to few cases which showed that there is deep space in the basic study and theory, technical, methods and information evaluation facilities matters. Therefore, it is recommended that the management information systems design with information needs certified in all hygiene ministry parts, are done by scientific methods.

In general, it is recommended that at first existence management information systems in the medical equipment office of medical sciences university should be evaluated with concentration on the university information management under vision of one unit or independent management information center, there being the possibility of management and omission of the managing parallel working in the different units of the university. Then, the second stage underlines the creation of expertise workgroups, information quantity based on certifying scientific methods and according to the integration management information system with the use of new technology of design.

**Acknowledgement**

The article is result of a searching design entitled “information producing process rectifying based on managers information needs of medical sciences universities of Hormozgan remedy hygiene services”, ratified by the medical sciences university. From the office’s respectful managers of MEOs of medical sciences universities of country, it was appreciated that they cooperated in running this plan.
